# Genome Sequence of *Clostridium diolis* Strain DSM 15410, a Promising Natural Producer of 1,3-Propanediol

**DOI:** 10.1128/genomeA.00542-13

**Published:** 2013-08-01

**Authors:** Yu Wang, Fei Tao, Hongzhi Tang, Ping Xu

**Affiliations:** State Key Laboratory of Microbial Metabolism and School of Life Sciences & Biotechnology, Shanghai Jiao Tong University, Shanghai, China

## Abstract

*Clostridium diolis* strain DSM 15410 is considered one of the best natural producers of 1,3-propanediol because of its appreciable substrate-tolerant ability, yield, and productivity. Here, we present a 5.85-Mb assembly of its genome sequence. We have annotated the coding sequences responsible for glycerol utilization and 1,3-propanediol fermentation.

## GENOME ANNOUNCEMENT

1,3-Propanediol (1,3-PD), one of the most important valuable platform chemicals, can be produced from renewable resources by using microorganisms (1). The application of 1,3-PD is mainly in the synthesis of polymers, such as polytrimethylene terephthalate (PTT), a new polyester with superior stretching and stretch recovery characteristics (2). Tremendous growth of the biodiesel industry has created a glycerol surplus, making it desirable to produce 1,3-PD from glycerol (2).

A number of microorganisms can ferment glycerol to 1,3-PD, such as strains of *Clostridium*, *Klebsiella*, *Citrobacter*, *Enterobacter*, *Lactobacillus*, and *Halanaerobium* (2–7). Among these microorganisms, nonpathogenic *C. diolis* may be preferred for the industrial production of 1,3-PD due to its appreciable substrate-tolerant ability, yield, and productivity (2, 8). *C. diolis* strain DSM 15410 (formerly *C. butyricum* DSM 5431) can produce 1,3-PD from glycerol anaerobically, and it has been studied for decades as a type strain (3, 9, 10). Previous studies have indicated that *C. diolis* DSM 15410 can produce 70.3 g liter^-1^ of 1,3-PD, with a yield of 0.68 mol mol^-1^ and a productivity of 1.5 g liter^-1^ h^-1^ (10). However, performance for the production of 1,3-PD is far below the optimum, mainly due to the formation of byproducts (11). Careful analyses of the pathway and kinetics of 1,3-PD synthesis are required to genetically modify the strain. Genome sequencing and bioinformatics will be of great help in this regard. For this purpose, we sequenced the genome of strain DSM 15410.

Here, we present the draft genome sequence of *C. diolis* strain DSM 15410, determined by using the Illumina HiSeq 2000 system, which was performed with a paired-end library by the Chinese National Human Genome Center, Shanghai, China. The reads for strain DSM 15410 were assembled into 177 contigs by using Velvet (12). Gene prediction and genome annotation were carried out by using the RAST annotation server (13). The G+C content was calculated by using the genome sequence.

The draft genome sequence of strain DSM 15410 is comprised of 5,853,914 bases with a GC content of 29.7%. There are 5,213 predicted coding sequences (CDS), together with 98 RNAs, in the genome sequence of strain DSM 15410. We have predicted 10 CDS responsible for glycerol and glycerol-3-phosphate uptake and utilization. We annotated the 1,3-PD operon, including the glycerol dehydratase and 1,3-PD dehydrogenase-encoding genes, which has a high level of identity (81% identity) with the 1,3-PD operon from *C. butyricum* strain VPI 1718 (14). The CDS responsible for byproduct formation, such as butyrate, lactate, and butanol, were also annotated. These CDS should be further investigated to eliminate side reactions and improve the efficiency of 1,3-PD production. Moreover, there are 78 CDS that have been annotated as antibiotics and toxic compound-resistance genes, while genes related to virulence, disease, and defense were not found.

### Nucleotide sequence accession numbers. 

The whole-genome shotgun project has been deposited at DDBJ/EMBL/GenBank under the accession number AQQG00000000. The version described in this paper is the first version, AQQG01000000.
